# Intravital microscopy in the study of the tumor microenvironment: from bench to human application

**DOI:** 10.18632/oncotarget.24957

**Published:** 2018-04-13

**Authors:** Emmanuel M. Gabriel, Daniel T. Fisher, Sharon Evans, Kazuaki Takabe, Joseph J. Skitzki

**Affiliations:** ^1^ Department of Surgery, Section of Surgical Oncology, Mayo Clinic, Jacksonville, FL 32224, USA; ^2^ Department of Immunology, Roswell Park Cancer Institute, Buffalo, NY 14263, USA; ^3^ Department of Surgical Oncology, Roswell Park Cancer Institute, Buffalo, NY 14263, USA

**Keywords:** intravital microscopy, vasculature, lymphocyte, trafficking

## Abstract

Intravital microscopy (IVM) is a dynamic imaging modality that allows for the real time observation of biologic processes *in vivo*, including angiogenesis and immune cell interactions. In the setting of preclinical cancer models, IVM has facilitated an understanding of the tumor associated vasculature and the role of effector immune cells in the tumor microenvironment. Novel approaches to apply IVM to human malignancies have thus far focused on cancer diagnosis and tumor vessel characterization, but have the potential to provide advances in the field of personalized medicine by identifying individual patients who may respond to systemically delivered chemotherapeutic drugs or immunotherapeutic agents. In this review, we highlight the role that IVM has had in investigating tumor vasculature and the tumor microenvironment in preclinical studies and discuss its current and future applications to directly observe human tumors.

## INTRODUCTION

Intravital microscopy (IVM) is an imaging technique that allows for the real time, direct observation of organic processes *in vivo*. In contrast to standard conventional microscopic interrogation of fixed tissue sections, IVM allows for a more comprehensive understanding of biologic processes as they occur in the host environment in real time. The dynamic capability of IVM offers several advantages over other traditional *in vitro* methodologies, such as histopathology, immunohistochemistry (IHC), or flow cytometry, that may only provide information in static snapshots of time. Additionally, unlike other live imaging techniques, such as computerized tomography (CT) scan or magnetic resonance imaging (MRI), IVM allows for high resolution observations at the cellular and subcellular level rather than the organ level [1–4].

The direct visualization of cell populations and their functions in various tissue microenvironments is a powerful tool to dissect complicated and dynamic biological interactions related to tissue metabolism [5, 6], remodeling [7, 8], angiogenesis [9, 10], inflammation [11, 12], and immunity [13, 14]. Moreover, the application of IVM to the study of carcinogenesis has advanced the understanding of several processes involved in the tumor microenvironment [15], including tumor angiogenesis [16], lymphocyte trafficking and infiltration [17], and tumor metastasis [18]. Thus, the greatest attribute of IVM in the study of human disease is the ability to examine biological functions in real time at the cellular level.

Although IVM is an older technique, it has evolved along with conventional microscopy, and thus has been combined with many different visualization methodologies, i.e., bright field, single photon fluorescent imaging, confocal microscopy and multiphoton microscopy [2–4, 19]. IVM has progressed significantly from its beginnings nearly 100 years ago examining the development of blood vessels during tumor formation in rabbit ears [20]. Contemporary IVM can employ multiphoton microscopy to allow for clear detection and quantification of multiple fluorescent molecules deep in tissue sites allowing for the discernment of the interactions between a variety of different cell types involved in biologic processes [21, 22]. Multiphoton IVM has emerged in this field as the detection of multiphoton events is limited to a narrowly defined area of the microscopy field, allowing for a deeper observation of tissues to approximately 100–1,000 μm in depth [23]. In contrast, traditional confocal microscopy is typically limited to tissue depths less than 100 μm.

However, it is important to note that the breadth of these advances have largely been achieved in preclinical animal models. To date, there are only a handful of reports demonstrating successful IVM techniques in the study of human cancers, though these pioneering studies highlight the potential of IVM to human disease [24–27]. In this brief review, we discuss the use of IVM to investigate two areas in cancer research: (1) tumor-associated vasculature and its implications on drug delivery and (2) the tumor microenvironment with emphasis of immune cell trafficking and interaction with tumor cells. We highlight the preclinical studies in these two areas where IVM has made significant contributions. Lastly, we present the investigations utilizing IVM in the study of human subjects with cancer. These nascent studies of IVM applied to human disease demonstrate the feasibility and utility of an approach that may lead to breakthroughs in the understanding of human carcinogenesis and anti-tumor therapies.

## IVM IN THE STUDY OF TUMOR VASCULATURE

Angiogenesis is a critical component of tumor growth and is an ideal process for the development of anti-cancer treatment [28–31]. As cancer cells multiply and divide, they require the formation of new blood vessels in order to obtain nutrients for continued growth [32–35]. Thus, tumor-associated angiogenesis is critical for tumor growth and development. Moreover, cancer cells can also use these vessels to gain access to the systemic circulation, which facilitate intravasation and distant metastases [36]. The importance of angiogenesis in cancer has been exploited by the development of targeted agents against tumor vessel formation. Bevacizumab is a vascular endothelial growth factor (VEGF) antagonist and was the first targeted agent for angiogenesis [37, 38]. There are other anti-angiogenic agents that are currently approved for use in certain countries or being actively investigated in clinical trials. These include endostatin, which is a broad-spectrum endogenous angiogenesis inhibitor [39], and angiostatin, which is an endogenous protein derived from plasminogen that inhibits angiogenesis by targeting vascular endothelial cell growth [40], thalidomide and immunomodulatory drugs (e.g., lenalidomide) which have antiangiogenic properties and inhibit the production of interleukin (IL)-6, which is a growth factor for the proliferation of myeloma cells and other cancers [41].

**Figure 1:**
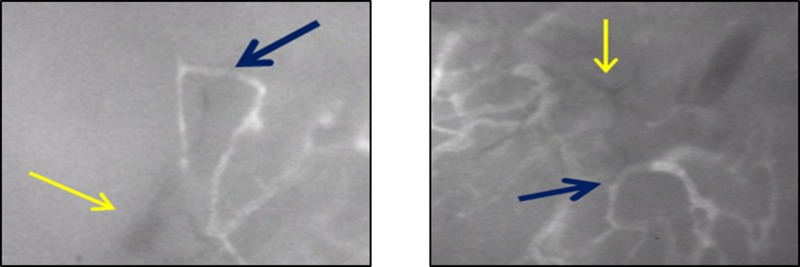
Examples of tumor associated blood vessels as observed in mouse by intravital microscopy (IVM) Tumor vessels are characterized by a haphazard, disorganized distribution, poorly defined branchpoints or multiple vessels intersecting into large, abnormally dilated vessels; and aberrant structural formations such as hairpin loops or circular patterns (blue arrows). Tumor vessels are also characterized by the absence of fluorescent dye uptake suggesting absent or non-detectable flow (yellow arrows).

Key in the development of anti-angiogenesis treatments is the number of differences between tumor-associated vasculature and normal, healthy vessels that have been described [2, 42–45]. Normal vessels are characterized by having a well-defined organization and distribution, non-dilated appearance, normal permeability, complete basement membrane, appropriate expression of endothelial and smooth muscle markers, and a physiologic rate of blood flow. The stromal tissue surrounding normal vessels also usually maintain a low interstitial pressure. In contrast, tumor vasculature is characterized by having a highly disorganized or haphazard distribution with vessels of various shapes and patterns, unusual dilated appearance, high permeability or “leakiness,” incomplete or absent basement membranes, abnormally high or low expression of surface markers, and a sluggish or absent rate of blood flow. Many of these characteristics of tumor blood vessels can be directly observed by IVM (Figure 1). In addition, tumor vessels exist within a high surrounding interstitial pressure, which has been termed interstitial hypertension. This interstitial hypertension has been shown to contribute to abnormal blood flow and may hinder the delivery of drugs into the tumor microenvironment [2, 46–49].

The dynamic nature of IVM has provided an understanding of the temporal events that are sequentially involved with tumor angiogenesis and highlighted the differences between normal vessels and tumor vessels. Although these differences have been examined with both conventional methodologies, such as IHC or *in vitro* models, there are major limitations in *ex vivo* models, including the inability to fully recapitulate the *in vivo* biologic architecture and blood flow, reproduce the various important signaling pathways that function in concert to generate angiogenesis, and reestablish the intrinsic biomechanical forces related to tumor vessels, such as interstitial hypertension and blood flow [17, 24, 50]. Conversely, IVM has allowed for the study of tumor angiogenesis during its multiple phases and has increased the understanding of this process in ways that conventional methods are not capable. Using different *in vivo* animal models, initial signs of tumor angiogenesis and abnormal tumor vasculature have been observed within 2–4 days following tumor implantation [51]. For example, in a heterotopic model of mouse glioma, these events included tumor vessel dilatation and abnormal architectural changes. At day 6 post-implantation, additional tumor vessel aberrancies were characterized, including altered permeability to red blood cells resulting in tumor-associated hemorrhage [10]. As tumors continue to grow, angiogenesis preferentially occurs toward the tumor periphery, whereas the central area becomes increasingly necrotic and less vascularized [52]. This is consistent with the general principles of tumor growth established by more conventional methods to study tumors, such as CT scan or histopathology. However, IVM has allowed for a more dynamic and directly observable temporal understanding of tumor angiogenesis over time. This has provided evidence for the different phases of angiogenesis, from early onset to the maturation of tumor vasculature.

The physical parameters of tumor vessel architecture and blood flow have also been extensively studied using IVM. These parameters include blood flow velocity, pressure gradients, and vessel shear, all of which have direct implications on tumor metabolism, oxygenation, immune cell interactions and trafficking, drug delivery, and even angiogenesis itself [53–55]. Following observation and direct capture of fluorescently-enhanced images and videos, post-observational analyses allow for these measurement determinations. Computer software facilitates the calculation of tumor vessel diameter, density, vessel permeability, vascular surface area, flow rates, and resistance [56, 57]. These abnormal values have been characterized and compared to those of normal, healthy vessels, [17, 24, 58] which has provided further insight into the role of tumor vasculature in carcinogenesis.

Based on these observations captured by IVM, investigators have analyzed drug delivery within tumor vasculature and the effects of anti-angiogenic agents on tumor response. Modeling of drug delivery has been significantly enhanced with IVM. Historically, transport of fluorescent molecules was characterized through both the intravascular and interstitial fluid compartments in animal tumor models [2, 6, 17, 43, 47, 54, 59, 60]. Many of the commercially available fluorochromes have molecular weights that are similar to chemotherapeutic agents, which suggests that the delivery of these fluorescent dyes approximate drug delivery. For example, fluorescein and indocyanine green (ICG) have molecular weights of 332 g/mol and 775 g/mol, respectively. These are similar to the molecular weights of various chemotherapeutic drugs, including cyclophosphamide (261 g/mol), oxaliplatin (397 g/mol), doxorubicin (544 g/mol), and irinotecan (587 g/mol). Some of these agents, such as doxorubicin, are intrinsically autofluorescent, which facilitates analysis of its delivery and diffusion through tumor vessels into the tumor interstitium [61].

From IVM observation, the permeability of tumor vessels can be calculated and compared to that of normal vessels. Permeability to macromolecules is critical to drug delivery reaching tumor cell targets through the tumor interstitium. Studies have shown that tumor vessels tend to have increased permeability or “leakiness” to macromolecules with molecular weights on the order of 150,000 to 500,000 g/mol, but that their diffusion is limited by interstitial pressure gradients across the tumor parenchyma and tumor vessels [46, 62]. Thus, novel strategies to optimize drug delivery through manipulation of tumor vessel blood flow and pressure gradients are important for generating anti-tumor responses and can be tested *in vivo* using IVM [17, 63].

Manipulations of the tumor vasculature and drug delivery continue to be developed and interrogated with IVM. Innovative drug delivery systems visualized by IVM utilizing nanoparticles to enhance tumor responses offer unique approaches to augment the efficacy of chemotherapeutic agents [16, 64, 65]. Tumor vessel responses to anti-angiogenic drugs such as bevacizumab can also be directly observed over time with IVM [55, 66], which have shown partial restoration of normal vessel permeability and interstitial fluid pressures that equate to enhanced anti-tumor responses. In summary, IVM has and continues to provide novel information regarding tumor vasculature, blood flow, and drug delivery in preclinical cancer models.

## IVM IN THE STUDY OF THE TUMOR MICROENVIRONMENT

Imaging with IVM has provided crucial information with regard to leukocyte migration and trafficking into lymph nodes [67, 68] and tumors [2, 17, 69]. The behavior of immune effector cells using IVM and preclinical tumor animal models *in vivo* has demonstrated biological differences from their behavior *in vitro*. Examples of such differences include mechanisms as to how T cells are activated to kill tumor cells and how leukocyte function-associated antigen-1 (LFA-1) plays a critical role in leukocyte rolling in vessels [13, 70–72]. A particular advantage of IVM technology is its ability to investigate cell-cell interactions, providing unique insight into the function of immune effector cells against tumor. Fluorescently labeled lymphocytes can be directly observed by IVM to traffic along tumor microvasculature, and various interventions can be tested in order to determine the optimal regional signals that support tumor infiltrating lymphocytes [17]. Similarly, myeloid derived immune cells including monocytes and macrophages have also been studied in the tumor microenvironment using IVM [15].

It has been well established that CD8+ effector T cells play a significant role in the adaptive tumor immunity response through their production of interferon-gamma (IFN-γ) and initiation of the Fas ligand-dependent cytolytic pathway following T cell receptor (TCR) interaction and activation with tumor antigen [73, 74]. The infiltration of CD8+ T cells into various tumor types have been correlated with improved clinical outcomes for patients with melanoma, breast cancer, and malignancies of gastrointestinal origin [75–80]. Lymphocyte trafficking is a critical precursor to infiltration into tumor as effector T cells need to home in to cancer sites in order to deliver their anti-tumor effects. Cell adhesion molecules, such as intracellular adhesion molecule-1 (ICAM-1) involved in lymphocyte firm adhesion to tumor vessels that precedes diapedesis into the tumor interstitium, have been shown to play important roles in lymphocyte trafficking [81]. Although transcriptional profiling studies have identified ICAM-1 and other surface molecules that influence T cell migration, one of the previous major gaps in the understanding of effector cell trafficking to the tumor microenvironment was determining whether these events could be manipulated in order to optimize tumor responses to effector cell function. Furthermore, the temporal sequence of events with respect to leukocyte trafficking and tumor infiltration has also been better characterized with IVM, highlighting another advantage of this technique.

**Figure 2:**
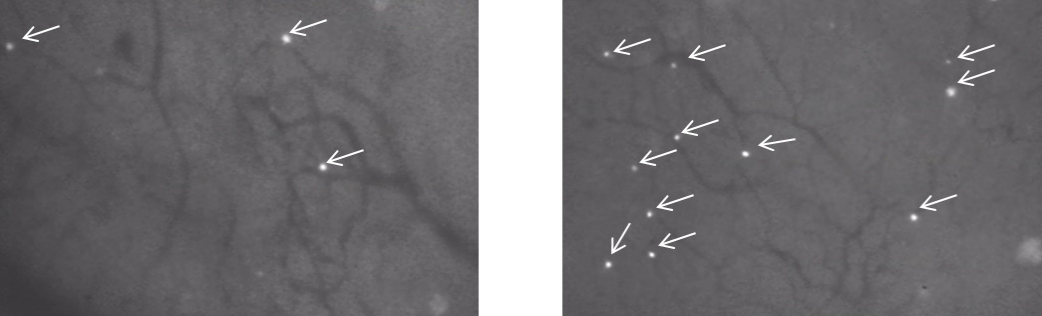
Examples of fluorescently labeled (fluorescein) lymphocytes trafficking to tumor in a mouse model as observed by IVM Lymphocyte behavior is captured in real time by IVM. Advantages of IVM in this tumor model include its high resolution to observe cell-to-cell interactions and to follow the number of events over time in response to different experimental or therapeutic interventions. White arrows highlight the infiltration of fluorescein labeled trafficking lymphocytes. In this case, the number of infiltrating lymphocytes was increased in response to a thermal stimulus. Left panel: before thermal stimulus; Right panel: after thermal stimulus.

Using IVM to track fluorescently labeled adoptively transferred lymphocytes, investigators have directly addressed these issues. Fisher *et al.* showed that effector T cell trafficking and infiltration into tumors was limited despite the presence of proinflammatory cytokines such as IFN-γ [17]. The application of a thermal stimulus resulted in increased signaling through the interleukin-6 (IL-6) pathway and modified the tumor vasculature with the end result of increased effector T cell trafficking and infiltration into target tumor, which could be directly observed and quantified in real time using IVM (Figure 2). Mechanistically, this was accomplished through a combination of increased selectin and ICAM-1 expression, promoting extravasation of effector and memory T cells in tumor tissue [17, 82]. A decrease in the infiltration of regulatory T cells (Tregs) was also noted, further favoring an anti-tumor response within the tumor microenvironment. Xu *et al.* later showed that IVM could be used to investigate other signaling molecules involved with T cell trafficking using a constructed photoactivable (PA) chemokine C-X-C motif receptor 4 (PA-CXCR4). CXCR4 along with other chemokines like CXCR3 has been shown to play an important role in lymphocyte trafficking and immunosurveillance [82–84]. Using a mouse melanoma model, the PA-CXCR4 construct was shown to transmit intracellular CXCR4 signals in response to a 505-nm light stimulus, resulting in activation of T cell polarization and migration to tumor [85]. This approach improved the efficacy of adoptive T cell transfer with a significant reduction in tumor growth *in vivo*. These findings suggested that the use of photoactivatable chemokine receptors influenced lymphocyte trafficking with outstanding spatial resolution in tissues. Similar to the study by Fisher *et al.* which targeted IL-6 signaling to improve the delivery of effector T cells into the tumor, the work by Xu *et al.* represents another strategy in optimizing T cell trafficking and infiltration to tumors, utilizing IVM as a means to demonstrate such observations.

In addition to lymphocyte trafficking and infiltration, IVM has also allowed for the study of tumor associated macrophages (TAMs). IVM has been used to elucidate various functions of TAMs *in vivo*, ranging from phagocytosis of antibody labeled tumor cells to interactions with tumor and other immune cells within the tumor microenvironment [86]. The latter function of TAMs has been observed with IVM at several *in vivo* locations, including the tumors themselves, tumor draining lymph nodes (TDLNs), and secondary lymphoid organs including the liver and spleen [15]. For example, studies utilizing IVM have shown that TAMs interact with T cells at high endothelial venules (HEVs) located in proximity to TDLNs, where tumor antigen presentation occurs [87, 88]. This interaction within TDLNs has been observed in as little as 8 hours following adoptive transfer of T cells and tumor antigen pulsed dendritic cells [89, 90]. The amount of interaction then increases substantially over the next several hours, forming a network of steady-state antigen presenting cells (APCs) for trafficking lymphocytes [91, 92]. Tumor-draining lymph nodes were also found to have diminished expression of CCL21, a chemokine critical for T cell trafficking resulting in decreased interactions between circulating T cells and lymph node vessels by IVM [93]. These studies have highlighted the unique use of IVM to characterize the temporal sequence of events involved in tumor antigen presentation and downstream migration of programmed T cells.

Using IVM, TAMs have also been observed to play important immunosuppressive roles at tumor sites [94]. In a study using a murine breast cancer model and multiphoton microscopy, TAMS were identified at the edges of tumors and shown to migrate in association with tumor growth at the periphery [95]. Interestingly, perivascular macrophages within the breast tumors were associated with tumor cell intravasation even in the absence of regional tumor angiogenesis, further supporting a role for TAM-promoting tumorigenesis and metastasis. Mechanistically, this pattern of co-migration between TAMS and breast tumor cells was dependent on macrophage-derived epidermal growth factor (EGF) and tumor cell-derived colony stimulating factor 1 (CSF-1), which were shown to create a paracrine signaling loop between the tumor cells and TAMs [95–98]. Impairment of these TAMS using clodronate liposomes was shown to significantly decrease tumor cell motility and intravasation, providing evidence that TAMS could be targeted in the tumor microenvironment and that this approach could be observed directly with IVM [99, 100]. Thus, these TAM-associated mechanisms of tumorigenesis represent novel targets for therapy, for which IVM offers a unique role in their testing. Recent preclinical studies have highlighted this potential where macrophages were imaged eliminating circulating tumor cells following antibody therapy [101].

Similar to the interaction of TAMS with tumor cells to promote local tumor growth, metastases originating from the tumor microenvironment have also been an area where IVM has provided new insight [18, 102]. Like previous studies examining the periphery of tumors, preclinical models utilizing IVM have shown that the tumor margin contains functional lymphatic vessels that may represent regions where lymphatic metastases can occur [103]. A number of studies using IVM have characterized the mechanistic signaling pathways required for the entry of tumor cells into the regional lymphatics. A prominent example of one signaling pathway involved in tumor intravasation was studied by Giampieri *et al.*, which comprised a transforming growth factor-β (TGF-β) signaling cascade promoting Smad4 and epidermal growth factor receptor (EGFR) mediated downstream activation in a breast cancer model [104]. Using a construct of Smad4 fused to green fluorescent protein (GFP), expression of TGF-β could be quantified, and this was directly associated with single tumor cell motility and intravasation into blood vessels as observed by IVM. Further work by this group and others have shown that blockade of TGF-β can abrogate TGF-β-mediated tumor motility and intravasation into blood vessels seen by IVM, although other mechanisms of tumor cell motility may persist and lead to intravasation into tumor lymphatics [105, 106]. IVM has even been used to show how neutrophils can promote liver metastasis through interactions with circulating tumor cells [107].

Thus, in addition to the study of tumor vasculature, IVM has enabled multiple advances in the study of the tumor microenvironment, specifically in regards to tumor and immune cell interactions. Effector cell trafficking can be directly observed, which provides an important platform to study novel therapies to increase or optimize effector trafficking with the goal of augmenting anti-tumor responses. Mechanisms of tumor cell intravasation into blood vessels or lymphatics, as observed by IVM in real time, have likewise presented opportunities to study the early events of metastasis and strategies to prevent them.

## HUMAN APPLICATIONS OF IVM

While IVM has been extensively utilized in preclinical animal models to study tumor vessels and the tumor microenvironment, the direct use of IVM in human subjects has been much less prevalent. However, novel applications of IVM have been reported and show promise for translational impact of IVM in the study of human diseases, particularly in regard to tumor vasculature and the tumor microenvironment. Specifically, IVM techniques have been applied to the endoscopic evaluation of gastrointestinal tumors (originating from the esophagus or colon), the cystoscopic evaluation of bladder urothelial cancer, and most recently to melanoma, including primary tumors or in-transit lesions, as summarized in Table 1. The superficial or endoluminal location of these tumor types facilitates direct observation with IVM and has helped to demonstrate the feasibility and applicability of IVM in human subjects.

**Table 1:** Applications of human intravital microscopy (HIVM)

Investigator^Reference^	Disease Site	Device	Feasibility	Differentiate normal tissue from pre-cancer/cancer
Kiesslich *et al.* [110]	Esophagus	Confocal laser endomicroscope (Pentax and Optiscan)	Yes	Yes
Nakao *et al.* [111]	Stomach	Confocal laser endomicroscope (Mauna Kea Technologies and Fujinon)	Yes	Yes
Wallace *et al.* [27]	Esophagus and Colon	Confocal laser endomicroscope (Mauna Kea Technologies)	Yes	Yes
Xie *et al.* [112]	Colon	Confocal laser endomicroscope (Pentax)	Yes	Yes
Wu *et al.* [119]	Bladder	Confocal laser endomicroscope (Mauna Kea Technologies)	Yes	Yes
Fisher *et al.* [24]	Skin (Melanoma)	Roswell Park Cancer Institute (RPCI) intra-operative microscope	Yes	Not performed

HIVM has been used to study human malignancies in a variety of organ sites. The surface or lumenal nature of these malignancies facilitates the interface between the microscopic objective and the tumor cells. Observations are feasible in real time and can often differentiate between normal versus abnormal tissue.

With regard to gastrointestinal endoluminal tumors, IVM has been directed to tumors of the esophagus or colon via endoscopy [108]. Parallel to *in vitro* observations, endoscopic imaging technology, or endomicroscopy, has been demonstrated in numerous studies to be feasible for the *in vivo* observation of cellular morphology of the mucosa in the upper and lower gastrointestinal tract at the cellular and subcellular level [109, 110]. Differences in nuclear and cellular morphology between normal and aberrant cells, enhanced with intravenous (IV) administration of fluorescein, have been strongly correlated with the diagnosis of neoplasia [27]. In the upper gastrointestinal tract, endomicroscopy distinguished between normal mucosa and Barrett's esophagus with sensitivity and specificity each over 90% [110]. Detection of early human gastric cancer has also been shown to be feasible, which in some instances has not required enhancement with IV fluorescein injection [111]. In the lower gastrointestinal tract, differentiation of neoplastic versus non-neoplastic colonic polyps has also been demonstrated with high sensitivity and specificity (greater than 90%) using endomicroscopy [112]. Furthermore, several studies have reported the feasibility of differentiating dysplastic cells from normal cells by conjugating fluorescent peptides to precancerous or cancer cells. This technique has further facilitated the detection of dysplastic areas by IVM in patients with either colon or esophageal malignancies confined to the mucosa [113–115].

For bladder urothelial tumors, IVM has similarly been directed to the bladder urothelium via cystoscopy [116]. The main function of IVM in this setting has been to predict which areas of the bladder urothelium are malignant. Small, reusable imaging probes ranging in diameter from 0.85 mm to 2.6 mm can be inserted into the working channels of standard cystoscopes [117] Based on fluorescence confocal microscopy in patients who received IV fluorescein, real time IVM of normal urothelium, carcinoma *in situ*, and low-grade and high-grade carcinoma have been correlated to results obtained with conventional histopathology [118, 119]. Using high-quality imaging and post-acquisition analysis, differences were categorized among the observed IVM images in relation to histopathological diagnoses. Interestingly, the ability to identify these different characteristics using IVM was consistent among novice and more expert urologists using cystoscopy, suggesting that IVM techniques may have a favorable learning curve [118]. Similar to the gastrointestinal tumors, fluorescently labeled antibodies specific to bladder tumor antigens, such as CD47, have also been employed to enhance the intravital imaging of urothelial tumors [25].

**Figure 3:**
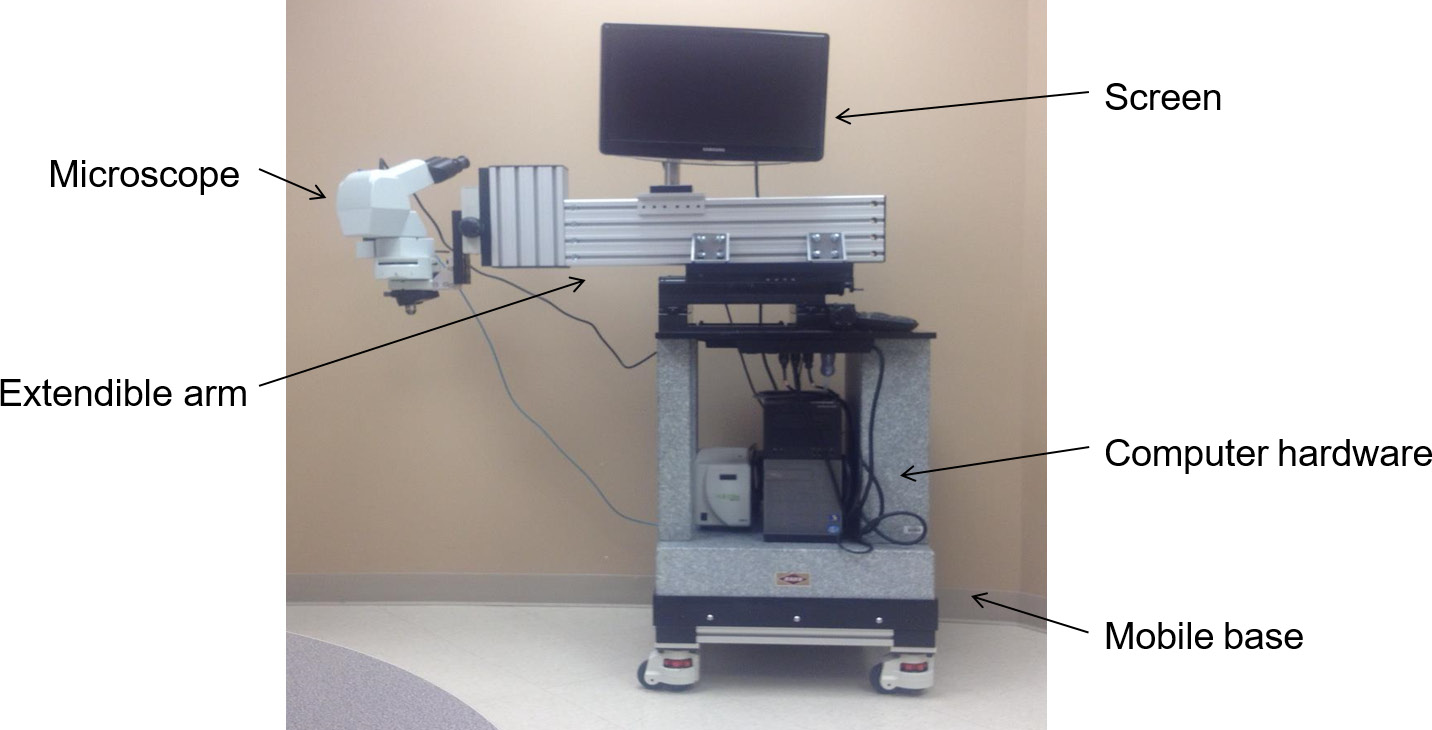
Depiction of the novel intravital microscope for human use Fisher *et al* have developed a portable, intravital microscope capable of interfacing with human tumors in real time during surgical resection in the operating room. The microscope and associated video microscopy hardware/software are wheel mounted to a solid granite base weighing over 360 kg, allowing for portability and stability during the IVM observation. The microscope objective lenses are placed on an extendable cantilevered arm to physically reach different anatomical areas of the patient.

**Figure 4:**
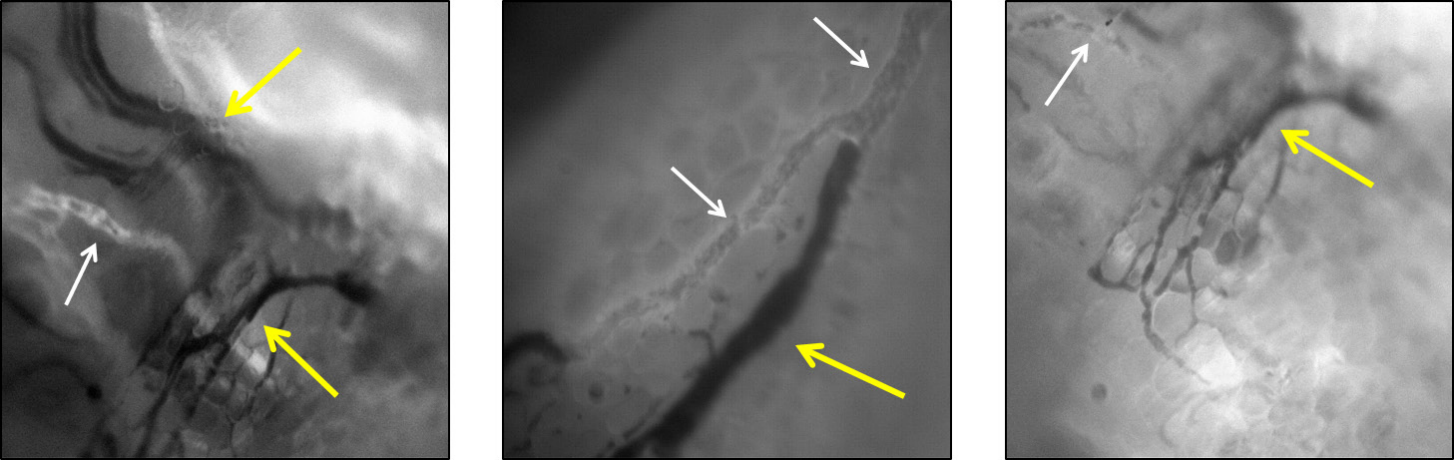
Examples of patient melanoma associated tumor vessels as directly observed by IVM in real time Interestingly, approximately 50% of these vessels are nonfunctional in that they do not have blood flow, supported by the observation that fluorescein is not present within the lumen of these vessels (yellow arrows). Normal appearing vessels are also present (white arrows). This new observation has implications for drug delivery to target tumor as patients with a high proportion of nonfunctional tumor associated vessels may have limitations in drug delivery and therefore drug efficacy.

**Figure 5:**
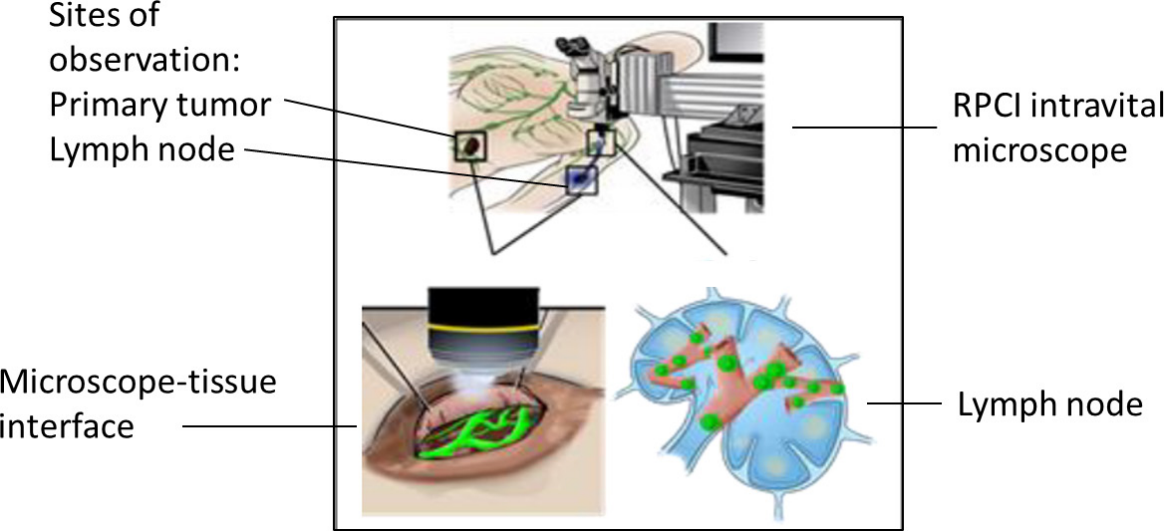
Schematic of ongoing clinical trial (NCT02857374), which is investigating the microvasculature associated with the primary tumor and the sentinel lymph node (SLN) of patients with melanoma who meet criteria for SLN biopsy In this illustration, the mobile intravital microscope interfaces with both the primary tumor (left lower panel) and the dissected SLN in the axilla (right lower panel). Nodal vessels and lymphatics are observed and correlated to node positivity.

In addition to these endoscopic and cystoscopic techniques, Fisher *et al* recently demonstrated the feasibility of IVM for patients with melanoma [24]. In this study, the authors used a patented, operating room (OR) compatible, mobile confocal microscope (Figure 3) in order to perform direct observation of patient tumor vasculature associated with primary melanoma or in transit disease. The aims of this clinical trial were to measure tumor diameter and blood flow delineated by IV fluorescein. Interestingly, up to 50% of tumor vessels did not support blood flow as determined by the absence of detectable fluorescein (Figure 4) [24]. To determine how these intraoperative observations compared with standard pathologic evaluation, tumor specimens from the same patients were analyzed to calculate vessel diameter, which showed that vessel diameters *in vivo* were on average twice as large as that found on IHC. Tissue shrinkage by pathologic preparation as well as the natural distention of vessels *in vivo* likely contributed to these differences in vessel size between real time IVM and pathologic measurements. Regardless of the reasons for this discrepancy, the larger size has important implications on blood flow and thereby on drug delivery that would be estimated based on histologic measurements of tumor vessel diameter and the previously discussed preclinical models. Following this clinical trial, this group is continuing the application of IVM to human subjects by performing real time, intraoperative observations of the vasculature associated with the sentinel draining lymph node (SLN) of patients with primary melanoma (NCT02857374) and in patients with primary peritoneal, fallopian tube, or ovarian cancer (NCT03297489). The human intravital microscope interfaces with the lymph node during the patients planned sentinel lymph node biopsy (Figure 5). Characteristics of the SLN associated vessels may have the future potential to serve as a biomarker for melanoma severity or prognosis when used in conjunction with other disease parameters. Similarly, the microscope interfaces with the peritoneal lining of patients with select peritoneal surface malignancies to evaluate tumor implant associated vessels.

This early study in human IVM for melanoma as well as the more established use of confocal microscopy in endoscopy and cystoscopy comprises the current applications of IVM in human patients. From these studies, advances have been made in the understanding of tumor vasculature and the diagnosis of endoluminal cancers. Further innovation with IVM holds promise to provide a greater understanding of human cancers and possibly optimization of responses to drug delivery.

## THE FUTURE OF IVM IN THE STUDY OF HUMAN CANCERS

Whereas the use of IVM has thus far been limited to observational and diagnostic applications, this innovative technology holds potential to further the understanding of human cancers and examine responses to chemotherapies and immunotherapies. While the first applications of fluorescent confocal microscopy were demonstrated in gastrointestinal mucosa and then duplicated for urothelial mucosa, the feasibility of IVM using an innovative intraoperative microscope has now opened a realm of possibility to investigate other primary malignancies. Some of the human cancers that may be amenable to IVM observation include inflammatory breast cancer or peritoneal surface malignancies or other etiologies, including colorectal or gastric cancers.

Inflammatory breast cancer is an aggressive form of invasive breast cancer and is characterized by tumor infiltration of the lymphatics resulting in an erythematous phenotype with dilated microvasculature [120, 121]. These characteristics make inflammatory breast cancer an ideal tumor type to be observed by IVM. This would allow the investigation of tumor associated blood vessels and lymphatics with respect to organization and blood flow. It may be reasonable to hypothesize that the nonfunctional proportion of vessels and lymphatics in inflammatory breast cancer may be similar to the 50% observed in melanoma by Fisher *et al*, and this would have important implications on drug delivery for this aggressive disease. Real time imaging of inflammatory breast cancer using IVM in humans has not yet been performed, but could be utilized during the delivery of neoadjuvant chemotherapy.

Similar to breast cancer, peritoneal carcinomatosis of gastrointestinal origin represents an additional surface malignancy that may be observed by IVM. Peritoneal carcinomatosis comprises a variety of tumors, which as a group are difficult to treat and typically are minimally responsive to systemic chemotherapy [122–124]. Cytoreductive surgery with hyperthermic intraperitoneal chemotherapy (CRS/HIPEC) may offer advantages over systemic treatment, though this has been associated with significant morbidity [125, 126]. Quantification of blood flow to peritoneal implants in real time can provide critical information on whether these tumors would be expected to respond to systemically delivered therapies. Nonfunctional vessels associated with peritoneal carcinomatosis would provide further rationale for the poor drug delivery and tumor response observed clinically.

Inherent to the use of IVM in any of these tumor types is the innovative application of this imaging technology to provide personalized precision medicine for patients with cancer [127]. For observable surface malignancies, determination of tumor vessel functionality can help guide individualized treatment by identifying which patients would be expected to sustain drug delivery. This could be particularly relevant for systemically delivered chemotherapy. Using inflammatory breast cancer as the example, the autofluorescent drug doxorubicin may be able to be tracked to tumor using human IVM technology. For drugs that are not intrinsically autofluorescent, fluorescein or ICG may be used as surrogates for drug delivery. Strategies to restore or enhance blood flow may also be tested in real time using IVM to optimize individualized drug delivery and response.

In addition to chemotherapy, immunotherapy can be investigated directly using IVM. Checkpoint inhibitors have comprised a major paradigm shift in the treatment of melanoma and are being investigated in many other cancers [128, 129]. As an immunotherapeutic agent, ipilimumab is a monoclonal antibody against cytotoxic T Lymphocyte Associated Antigen-4 (CTLA-4), the expression of which is mechanistically associated with anergy to tumor-associated antigens [130]. Targeted blockade of CTLA-4 led to unprecedented responses in metastatic malignant melanoma [131]. Treatment with pembrolizumab, an antibody against the programmed cell death 1 (PD-1) receptor, has been even more successful than ipilimumab and is associated with a more favorable toxicity profile, thus supplanting ipilimumab as the first line agent for patients with metastatic melanoma [132]. However, durable responses with these agents are still limited, and many patients develop resistance to these therapies [133]. To address these individualized patient issues, IVM may be used to demonstrate trafficking and infiltration of fluorescently labeled, adoptively transferred effector cells to tumor targets in order identify patients with the potential to respond to immunotherapeutics. Consistent with the emerging realm of personalized medicine, IVM may be able to select patients who will respond to different anti-tumor therapies in order to provide the most effective treatments.

In conclusion, many advances in the understanding of tumor vasculature and immune cell functions in the tumor microenvironment have been made using IVM, largely in preclinical models. While most of the applications of IVM to human malignancies have involved the detection of tumor cells or the observation of tumor associated vessels, current innovations offer the potential to expand the clinical use of IVM. These future novel directions for IVM may have important implications for personalized medicine and include the study of tumor vessel modulation to augment drug delivery and the role of fluorescently labeled immune effector cells in human tumors.

## Abbreviations

ICG: indocyanine green
IVM: intravital microscopy
PA: photoactivable
SLN: sentinel lymph node
TAM: tumor associated macrophages
TDLN: tumor draining lymph node
Treg: regulatory T cell.
